# Serum free light chain levels and renal function at diagnosis in patients with multiple myeloma

**DOI:** 10.1186/s12882-018-0962-x

**Published:** 2018-07-13

**Authors:** Punit Yadav, Paul Cockwell, Mark Cook, Jennifer Pinney, Hannah Giles, Yu Sandar Aung, David Cairns, Roger G. Owen, Faith E. Davies, Graham H. Jackson, J. Anthony Child, Gareth J. Morgan, Mark T. Drayson

**Affiliations:** 1 Department of Renal Medicine, University Hospital Birmingham NHS Foundation Trust, Birmingham, UK; 2 Institute of Immunology and Immunotherapy, University of Birmingham, Birmingham, UK; 3 Department of Haematology, University Hospital Birmingham NHS Foundation Trust, Birmingham, UK; 4 Clinical Trials Research Unit, University of Leeds, Leeds, UK; 5 Department of Haematology, Leeds Teaching Hospitals NHS Trust, Leeds, UK; 6 Myeloma Research Centre, Division of Molecular Pathology, The Institute of Cancer Research, London, UK; 7 Myeloma Institute of Research and Therapy, University of Arkansas for Medical Sciences, Little Rock, AR USA; 8 Department of Haematology, University of Newcastle, Newcastle-upon-Tyne, UK; 9 Clinical Immunology Service, College of Medical and Dental Sciences, University of Birmingham, Edgbaston, Birmingham, B15 2TT UK

**Keywords:** Myeloma, Serum free light chain level, Renal impairment

## Abstract

**Background:**

Renal impairment (RI) is common in multiple myeloma (MM) and is associated with poor survival. This study reports the associations between renal function and disease characteristics including serum free light chain (FLC) level at diagnosis in patients with MM.

**Methods:**

Using data from the Medical Research Council Myeloma IX trial, a multicentre, randomized, open-label, phase III and factorial-design trial, we assessed the relationships between renal function, demographic, and disease characteristics, including serum FLC levels, in 1595 newly diagnosed MM patients. Multivariable linear regression was utilised to identify factors that were associated with renal function at diagnosis. A receiver operating characteristic curve (ROC) was used to identify the optimal threshold for serum FLC level at diagnosis to predict severe RI.

**Results:**

52.8% of patients had an estimated glomerular filtration rate (eGFR) ≥60 ml/min/1.73 m^2^ (no RI), 37.3% an eGFR 30–59 ml/min/1.73 m^2^ (mild to moderate RI), and 9.8% an eGFR < 30 ml/min/1.73 m^2^ (severe RI). In a multivariable analysis, factors independently and negatively associated with eGFR at diagnosis were: higher serum FLC level, female gender, and older age. Elevated serum FLC level at diagnosis, irrespective of the paraprotein type, was strongly associated with severe RI. Receiver operating characteristic curve analysis showed a serum FLC level of > 800 mg/L as the optimal cut-off associated with severe RI (area under curve 0.86, 95% confidence interval 0.77–0.84).

**Conclusion:**

There was a strong relationship between higher serum FLC levels at diagnosis and the severity of RI that was irrespective of the paraprotein type. We report an increased risk of severe RI in patients presenting with serum FLC levels above 800 mg/L at diagnosis.

## Background

Renal impairment (RI), as defined by an estimated glomerular filtration rate (eGFR) of less than 60 ml/min/1.73m^2^, is present in up to 50% of patients with multiple myeloma (MM) and is associated with a poor prognosis [1, 2]. Renal impairment in MM can be multi-factorial; long-standing RI can be attributed to age-related co-morbidities such as hypertension and diabetes mellitus and is largely irreversible [3, 4]. Acute kidney injury (AKI) can be secondary to concurrent infections, dehydration, hypercalcaemia and use of nephrotoxic drugs and is potentially reversible with timely supportive care. The commonest cause of severe RI at diagnosis (estimated glomerular filtration rate (eGFR) < 30 ml/min/1.73m^2^) is through nephrotoxicity of the secreted immunoglobulin free light chain (FLC); this can be potentially reversed by the rapid lowering of the involved FLC with effective anti-myeloma therapy [5–7].

Up to one in five patients with MM have severe RI at diagnosis, this is ten-fold the prevalence of severe RI in an age- and gender-matched population from which MM arises [8, 9]. Approximately 4% of patients have RI that requires dialysis treatment [10]. Patients with MM and severe RI have a risk of death that is at least two-times higher than patients with MM and normal renal function [11].

Renal biopsy series from patients with MM have been restricted to individuals with severe RI, often requiring dialysis, and up to 90% of patients in these series have myeloma cast nephropathy (MCN) [6]. Myeloma cast nephropathy occurs because in some patients the FLC secreted by the neoplastic plasma cells are both at a high level and are nephrotoxic. Renal biopsies are rarely carried out in patients who do not have severe RI. It is hence not known what proportion of the mild to moderate RI in MM at diagnosis is attributed to nephrotoxicity of the secreted FLC, to other myeloma-related causes of renal damage or to the longstanding RI that develops due to other unrelated comorbidities. Outlining the relationship between the serum FLC level and renal function is important, as it has the potential to provide a mechanistic basis for the development of RI which may in turn direct future changes in treatment in MM based on the FLC levels and renal function at presentation.

In this study, we assessed the relationship between serum FLC levels and renal function across all stages of RI, except for patients receiving or likely to require dialysis, and analysed for a threshold level in serum above which there is a greater likelihood of FLC induced nephrotoxicity in patients with MM. We utilised the Medical Research Council (MRC) Myeloma IX trial dataset and included patient demographics and disease parameters, with a focus on paraprotein types and serum FLC levels at diagnosis, and renal function as measured by eGFR.

## Methods

The MRC Myeloma IX trial was a multicentre, randomized, open-label, phase-III, and factorial-design trial conducted in the United Kingdom (International Standard Randomised Controlled Trial Number 68454111). Trial protocol details have been published previously [12–15]. In brief, patients aged 18 years or older with symptomatic new MM were eligible to participate in the study. Exclusion criteria included pregnancy, asymptomatic MM, solitary bone plasmacytoma or extramedullary plasmacytoma, previous or concurrent active malignancies, and presence of severe AKI unresponsive to up to 72-h of rehydration, characterised by a serum or plasma creatinine more than 500 μmol/L, a urine output less than 400 ml/day, or a requirement for dialysis. A multicentre research ethics committee and local ethics committees approved the protocol and all patients gave written informed consent in accordance with the Declaration of Helsinki.

All patients recruited in the intensive and non-intensive treatment arms of the MRC Myeloma IX trial were eligible to participate in this study if blood samples had been sent for central laboratory analysis at entry to the trial. The two induction regimens: oral cyclophosphamide, thalidomide, and dexamethasone (CTD) treatment were compared with infusional cyclophosphamide, vincristine, doxorubicin, and dexamethasone (CVAD) in patients on intensive treatment arm. Patients in the non-intensive treatment arm were randomised to receive either melphalan and prednisolone versus attenuated CTD. The variables that were measured at diagnosis included: age, gender, serum creatinine (Roche®), paraprotein type (Sebia®), paraprotein level, and serum FLC level (Freelite® assays from The Binding Site Ltd., Birmingham, UK). The eGFR was calculated using the Modification of Diet in Renal Disease (MDRD) formula: eGFR (ml/min/1.73 m^2^) = 186 x (Serum creatinine / 88.4)^-1.154^ x (Age)^-0.203^ x (0.742 if female gender) x (1.210 if black ethnicity). Patients were divided into three categories based on their eGFR at diagnosis; these categories were based on those used in clinical practice for the classification of chronic kidney disease (CKD): no RI (eGFR ≥60 ml/min/1.73 m^2^), mild to moderate RI (eGFR 30–59 ml/min/1.73 m^2^) and severe RI (eGFR < 30 ml/min/1.73 m^2^). Renal biopsy data was not collected as part of the MRC Myeloma IX study protocol; hence results of biopsy findings if performed for any patient recruited in the study were unavailable for analysis.

Statistical analysis was performed using SPSS® for Windows, version 21.0 (SPSS Inc., Chicago, IL) and Graph Pad Prism 5.0 (GraphPad Software Inc., San Diego, CA). Categorical variables were summarised as frequencies and percentages. Comparisons for categorical variables among different groups were made with the chi-square test and Fisher’s exact test where appropriate. Continuous variables were expressed as mean and standard deviation (SD) if the data were normally distributed or median with interquartile range (IQR) for non-normally distributed data. Kruskal-Wallis test was used to compare differences in age, involved kappa FLC level, involved lambda FLC level, involved FLC level as per paraprotein type and eGFR at diagnosis by eGFR categories. Mann-Whitney *U* test was used to compare the differences in eGFR by gender and treatment arms at diagnosis based on eGFR categories. Differences in the involved serum FLC level at diagnosis for the two FLC isotypes by eGFR categories were also compared by the Mann-Whitney *U* test. Association between serum FLC level categories and eGFR categories was assessed by the chi-square test. The association between FLC level in serum and severe RI was evaluated by the receiver operating characteristic (ROC) curve analysis. The area under the curve (AUC) of sensitivity was plotted against 1-specificity and was reported with a 95% confidence interval (CI). ROC curve analysis was used to determine the optimal cut-off point that maximized the threshold value with the highest specificity and sensitivity for severe RI. Spearman’s correlation coefficient (r_s_) was calculated to evaluate the relationship between age and log_10_ transformed FLC and eGFR. A multivariable linear regression analysis was performed with factors that were independently associated with eGFR at diagnosis.

## Results

### Patient demographics and renal function

**Fig. 1:**
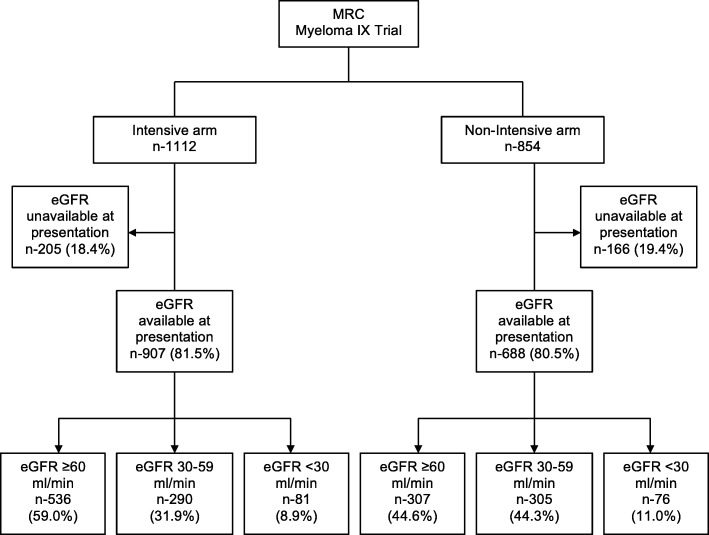
CONSORT diagram showing patient distribution by renal function. (MRC, Medical Research Council)

1966 patients with newly diagnosed symptomatic MM were recruited in the MRC Myeloma IX trial; 1112 (56.6%) were assigned to the intensive arm and 854 (43.4%) to the non-intensive arm of the trial (Fig. 1). Renal function from samples sent for central laboratory analysis at diagnosis were available in 1595 patients who were the focus of attention of this study. Out of these 1595 patients, 907 patients were from the intensive arm and 688 patients from the non-intensive arm. The remaining 371 patients had no samples sent for central laboratory analysis at diagnosis and were excluded from analysis in this study.

Table 1 shows the baseline patient characteristics. There was a significant association between age at diagnosis and the severity of RI (*P* < 0.001); patients with RI were older compared to patients with no RI. There was no difference in median eGFR by gender in patients presenting with mild to moderate (*P* = 0.49) or severe RI (*P* = 0.74), however, males had a higher eGFR compared to females in the no RI group (*P* = 0.006).

**Table 1:** Patient demographics at diagnosis

Variables	All patients (*n*-1595)	Patients with eGFR ≥60 ml/min (*n*-843)	Patients with eGFR 30–59 ml/min (*n*-595)	Patients with eGFR < 30 ml/min (*n*-157)	*P* value
Median eGFR at diagnosis (ml/min/1.73 m^2^) (IQR)	61 (45–79)	78 (68–92)	48 (40–54)	21 (16–25)	< 0.001
Treatment arm					
Intensive (%)	907 (56.9)	536 (63.6)	290 (48.7)	81 (51.6)	< 0.001
Non-intensive (%)	688 (43.1)	307 (36.4)	305 (51.3)	76 (48.4)	
Median age - years (IQR)	66 (58–73)	64 (57–70)	67 (60–75)	67 (60–73)	< 0.001
Gender					
Male (%)	959 (60.1)	531 (62.9)	342 (57.4)	86 (54.7)	0.04
Female (%)	631 (39.5)	311 (36.9)	250 (42.0)	70 (44.6)	
Unknown (%)	5 (0.3)	1 (0.1)	3 (0.5)	1 (0.6)	
Myeloma Type					
IgG (%)	977 (61.2)	534 (63.3)	371 (62.3)	72 (45.8)	< 0.001
IgA (%)	347 (21.7)	194 (23.0)	130 (21.8)	23 (14.6)	
IgM (%)	9 (0.5)	8 (0.9)	–	1 (0.6)	
IgD (%)	30 (1.9)	13 (1.5)	12 (2.0)	5 (3.2)	
LCO (%)	215 (13.5)	83 (9.8)	76 (12.8)	56 (35.7)	
Non-secretory myeloma (%)	17 (1.1)	11 (1.3)	6 (1.0)	–	
FLC type					
Kappa (%)	1029 (65.2)	568 (68.3)	373 (63.3)	88 (56.1)	0.006
Lambda (%)	549 (34.8)	264 (31.7)	216 (36.7)	69 (43.9)	
Involved FLC level (mg/L)					
Median Kappa FLC (IQR)	391.20 (79.51–1106.00)	224.10 (36.96–632.20)	474.60 (115.20–1427.00)	2217.00 (668.30–5424.00)	< 0.001
Median Lambda FLC (IQR)	503.00 (90.65–1848.00)	266.90 (57.11–942.10)	722.50 (151.40–2022.00)	3071.00 (1181.00–9079.00)	

Overall a higher number of patients were recruited in the intensive arm compared to the non-intensive treatment arm, this was more evident in patients presenting with no RI at diagnosis compared to those who presented with mild to moderate RI or severe RI (*P* < 0.001). Patients entering the intensive arm with no RI had a higher eGFR at diagnosis compared to those entering the non-intensive arm with no RI (*P* < 0.001). There was no significant difference in eGFR at diagnosis between the treatment arms in patients presenting with mild to moderate RI (*P* = 0.86) or severe RI (*P* = 0.09).

### Paraprotein and FLC isotype distribution and eGFR

Overall IgG myeloma was the predominant paraprotein type, however, the proportion of patients with light chain only (LCO) myeloma increased with worsening eGFR category. The median eGFR was lower for patients with LCO myeloma and IgD myeloma compared to those with other paraprotein types (LCO myeloma 49.0 ml/min/1.73 m^2^ [IQR 28.0–80.0]; IgD myeloma 49.0 ml/min/1.73 m^2^ [IQR 36.7–83.0]; IgG myeloma 62.0 ml/min/1.73 m^2^ [IQR 47.0–79.0]; IgA myeloma 64.0 ml/min/1.73 m^2^ [IQR 49.0–80.0]; and IgM myeloma 65.0 ml/min/1/73 m^2^ [IQR 60.5–84.5]) (*P* < 0.001). There was no significant difference in the median age of patients between the paraprotein types (*P* = 0.220). An involved kappa FLC isotype was more common than an involved lambda FLC. The median eGFR in patients with an involved lambda FLC isotype (58 ml/min/1.73 m^2^ [IQR 42–77]) was lower at diagnosis compared to patients with an involved kappa FLC (63 ml/min/1.73 m^2^ [IQR 47–80]) (*P* = 0.008).

### Serum FLC level and eGFR

**Fig. 2:**
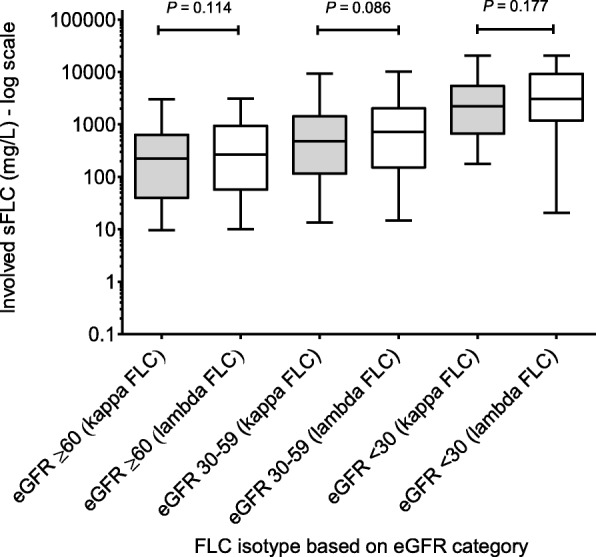
Serum FLC level distribution by eGFR. (showing a progressive rise in serum FLC level with worsening of eGFR category irrespective of the FLC isotype. Data presented as box plot with whiskers and solid line represents median)

**Fig. 3:**
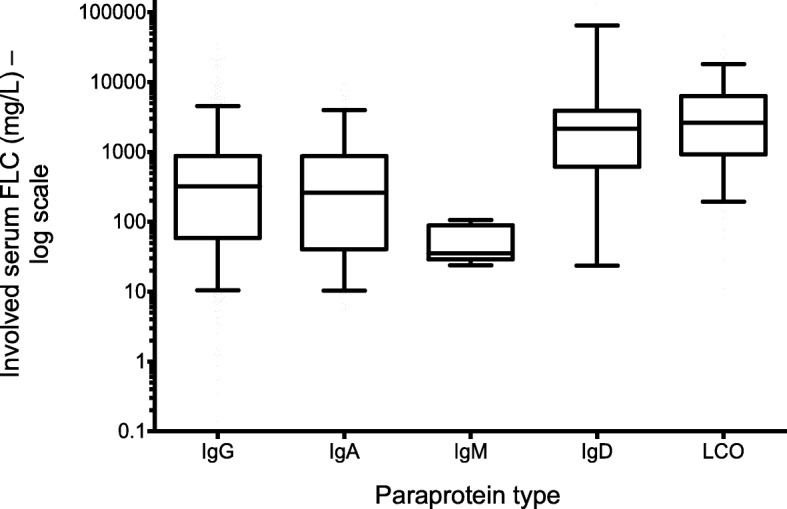
Involved serum FLC level by paraprotein type. (data presented as box plot with whiskers where solid line represents median)

There was a sequential rise in the median involved serum FLC level by eGFR category, irrespective of the FLC isotype. There was no significant difference in the median involved FLC level at diagnosis between the FLC isotypes for any of the three eGFR categories (Fig. 2). The median involved serum FLC level for patients with LCO myeloma and IgD myeloma was significantly higher compared to patients with other paraprotein types (*P* < 0.001) (Fig. 3), consistent with the lower eGFR seen in these patients.

**Fig. 4:**
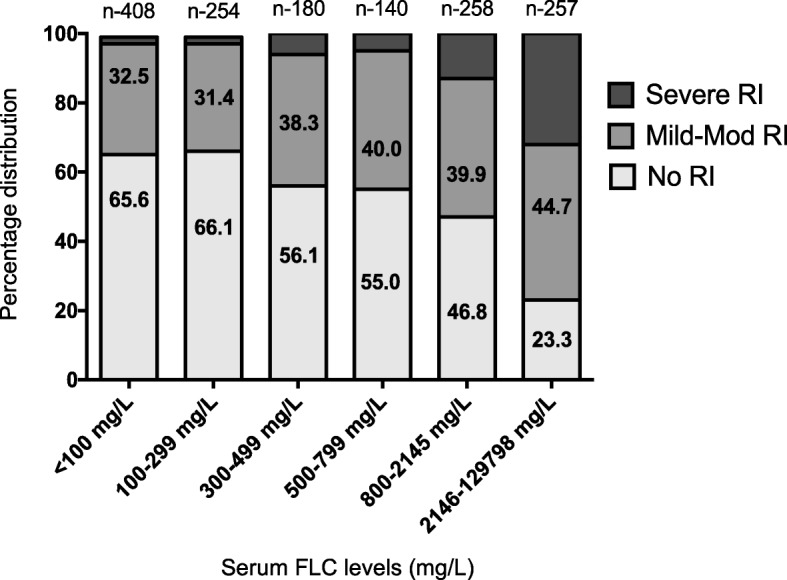
eGFR distribution across serum FLC level categories. (depicting increase in proportion of patients presenting with severe RI as serum FLC level at diagnosis rises above 800 mg/L)

In order to study the relationship of the varying levels of FLC in serum with renal function, we grouped patients on the basis of their eGFR at diagnosis into different serum FLC level categories (Fig. 4). On comparing patients with a serum FLC level < 100 mg/L with those with a serum FLC level of 100–299 mg/L we found no difference in the proportions that had mild to moderate RI or severe RI (*P* = 0.82). For patients with a serum FLC level of 300–499 mg/L the proportion of patients with mild to moderate RI increased by approximately 6% and for severe RI by approximately 4%. There was no further significant increase in the distribution of RI in patients with a serum FLC level of 500–799 mg/L (*P* = 0.94). However, as the serum FLC level increased > 800 mg/L there was an exponential increase in the percentage of patients presenting with severe RI in comparison to those with a serum FLC level < 800 mg/L (*P* < 0.001).

**Fig. 5:**
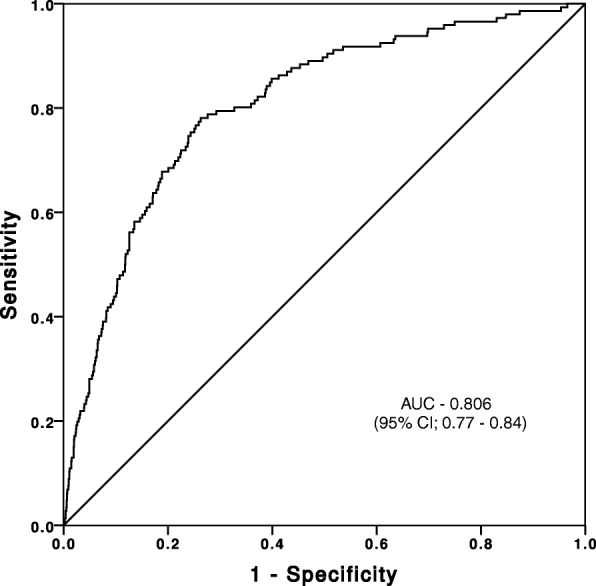
Receiver operating characteristic (ROC) curve testing association between FLC level and eGFR < 30 ml/min/1.73m^2^. (AUC, area under curve; CI, confidence interval)

We tested the association between serum FLC level at diagnosis and severe RI by calculating the composite score for sensitivity and specificity as determined by the ROC curve analysis. The AUC was 0.80 (95% CI 0.77–0.84; *P* < 0.001), indicating good predictive value for an eGFR < 30 ml/min/1.73 m^2^ (Fig. 5). A serum FLC level of 500 mg/L, the level recommended by consensus for the consideration of AKI due to MCN, was associated with a sensitivity of 84% and specificity of 60%. However, the optimal FLC cut-off for prediction of eGFR < 30 ml/min/1.73 m^2^ was 800 mg/L which had a sensitivity of 80% and a specificity of 70%.

### Correlations and regression modeling

There were significant correlations between age (r_s_ − 0.174; *P* < 0.001) and log_10_ transformed serum FLC level (r_s_ − 0.328; *P* < 0.001) with eGFR at diagnosis. Multivariable linear regression modeling incorporating all factors that were associated with eGFR at diagnosis showed a significant independent association with log_10_ transformed serum FLC level, age, and female gender but not for the paraprotein type or the FLC isotype (Table 2).

**Table 2:** Factors associated with eGFR at diagnosis

Factors	Multivariable linear regression			
		95% confidence intervals		
	Beta	Lower	Upper	*P* value
Age	−0.277	−0.478	−0.077	0.007
Log_10_ serum FLC level	−13.305	−15.449	−11.162	< 0.001
Female gender	−3.271	0.523	6.019	0.02
IgA Myeloma	0.406	−2.881	3.693	0.80
IgM Myeloma	7.802	−13.357	28.961	0.47
IgD Myeloma	−0.359	−10.203	9.486	0.94
LCO Myeloma	1.855	−1.663	5.373	0.30
Kappa FLC isotype	1.198	−1.634	4.029	0.40
Intensive arm	3.136	−1.023	7.295	0.13

(Male gender, IgG myeloma, and non-intensive arm are reference variables)

## Discussion

The purpose of this study was to investigate the relationship between renal function, demographic and myeloma characteristics, with a focus on serum FLC levels, in newly diagnosed patients with MM. Using data collected for the MRC Myeloma IX trial, we confirm a direct relationship between the serum FLC level and the severity of renal impairment. The risk of severe RI as defined by an eGFR < 30 ml/min/1.73 m^2^ at diagnosis, only became substantial when the involved serum FLC level was > 800 mg/L.

Renal impairment at diagnosis is an important, potentially modifiable risk factor in patients with MM [16]. In the MRC Myeloma IX trial, patients requiring dialysis or at a high-risk of requiring dialysis were excluded from recruitment. Despite this 47% of patients recruited in the study had an eGFR < 60 ml/min/1.73m^2^.

There are no kidney biopsy series that have systematically reported findings from newly diagnosed MM patients with mild to moderate RI. Thus, in most patients with MM it is not known to what proportion the mild to moderate RI at diagnosis is attributable to a) nephrotoxicity of FLC secreted by the myeloma clone, b) other myeloma-related causes of RI, and/or c) longstanding and largely irreversible RI due to unrelated comorbidities. In this study, the proportion of patients with mild to moderate RI at diagnosis was 37.3%, which compares to a CKD prevalence of 25% for age- and gender-matched populations from which these MM patients were recruited [9, 17]. This is consistent with two-thirds of the mild to moderate RI in this cohort being of longstanding origin and unrelated to the new diagnosis of MM. On assessing patients with mild to moderate RI for serum FLC level at diagnosis we found mild to moderate RI in 32.5% of patients with a serum FLC level < 100 mg/L, 31.4% with a serum FLC level 100–299 mg/L and 38.3% with a serum FLC level 300–499 mg/L. Only at a serum FLC level > 2146 mg/L was there a significant increment in the proportion of patients (44.7%) with mild to moderate RI (*P* < 0.001) (Fig. 4). We interpret these findings as indicating that only around 12% of MM patients with mild to moderate RI may have had RI secondary to FLC nephrotoxicity and in the remaining patients, RI was most likely due to other causes.

In patients with MM and severe RI at diagnosis, renal biopsy studies have shown that the predominant lesion in up to 90% of patients is MCN, which is a direct consequence of excess monoclonal FLC production [18]. The percentage of patients presenting with severe RI in the current study was approximately five-fold the reported prevalence of severe RI in an age- and gender-matched adult population [8, 9]. This is consistent with most of the severe RI observed in this study being attributable to MM rather than any pre-existing unrelated comorbidities. Also, all patients presenting with RI at MM diagnosis were investigated and treated for other myeloma-related causes of RI, such as dehydration and hypercalcaemia, prior to their recruitment in the MRC Myeloma IX trial.

FLCs have differential nephrotoxicity. In studies that have reported on MCN, the FLC level in serum associated with the lesion can range by 100-fold; conversely, some patients have no RI despite very high levels of FLC. The current study reinforces this observation; 19 (2.2%) of the 843 patients with no RI had levels of serum FLC above 5000 mg/L. Animal models and some previous clinical reports also indicate that MCN can develop in patients at far lower serum FLC levels function [19], indicating that nephrotoxicity of FLC varies greatly between patients.

LCO myeloma in the current study was associated with a lower eGFR compared to other paraprotein isotypes; this was specifically related to the serum FLC level and is not a function of the paraprotein isotype. This finding is consistent with a previous study, which reported higher urinary FLC levels in patients with LCO myeloma compared to patients with IgG myeloma and IgA myeloma [20]; in that study, RI was directly related to the urinary FLC levels. IgD myeloma was found in 1.8% of patients in the current study and the association of RI with elevated serum FLC levels at diagnosis was present in this group of patients as well. This and the possibility that IgD myeloma may still be diagnosed later than other myeloma types is under investigation in a larger cohort of patients.

78.4% of patients with LCO myeloma had a serum FLC level > 800 mg/L and 68.2% of these had RI. In comparison, only 27.2% of patients with an intact paraprotein MM had a serum FLC > 800 mg/L and 63.2% of these patients had RI, further illustrating that the relationship with renal function is related to the FLC level in serum and not the paraprotein type. This finding was also confirmed in a multivariable linear regression model where serum FLC level was the factor with the strongest association with eGFR; this relationship was independent of age and gender at diagnosis.

This study has a few limitations: in 371 patients no samples for renal function tests were sent for central laboratory analysis and therefore these patients had to be excluded from this study. Also, patients receiving or at high risk of requiring dialysis were not recruited, as a result, the association between FLC induced nephrotoxicity and serum FLC level could not be evaluated in dialysis patients. Renal biopsy data were not available and hence we were unable to correlate serum FLC levels with myeloma cast nephropathy and other renal pathologies. Lastly, these results are only applicable to the Freelite® assay and have not been validated in the other available FLC assays [21, 22].

## Conclusions

In conclusion, we report a strong relationship between higher serum FLC levels at diagnosis and the severity of RI, with a greater sensitivity and specificity for an FLC level > 800 mg/L with severe RI, irrespective of the paraprotein type. Further studies are now required to more accurately evaluate the impact of the early reduction of high serum FLC levels on RI in patients with MM, and to assess if this is associated with better long-term renal function and overall survival.

## Abbreviations

AKI: Acute Kidney Injury
AUC: Area under curve
CI: Confidence interval
CKD: Chronic kidney disease
FLC: Free light chain
Ig: Immunoglobulin
IQR: Interquartile range
LCO: Light chain only
MCN: Myeloma cast nephropathy
MM: Multiple myeloma
MRC: Medical research council
RI: Renal impairment
ROC: Receiver operating curve
r^s^: Spearmans coefficient
SD: Standard Deviation
